# Social Integration, Daily Discrimination, and Biological Markers of Health in Mid- and Later Life: Does Self-Esteem Play an Intermediary Role?

**DOI:** 10.1093/geroni/igaa026

**Published:** 2020-07-06

**Authors:** Jeffrey E Stokes

**Affiliations:** Department of Gerontology, John W. McCormack Graduate School of Policy and Global Studies, University of Massachusetts Boston

**Keywords:** Emotion, Health, Psychosocial, Well-being

## Abstract

**Background and Objectives:**

This cross-sectional study examines associations of social integration and daily discrimination with 4 biological markers of inflammation and cardiovascular health and tests whether self-esteem may mediate any of these effects.

**Research Design and Methods:**

Data came from 746 participants of the National Survey of Midlife Development in the United States (MIDUS) Refresher (2011–2014) and MIDUS Refresher Biomarker Project (2012–2016). Structural equation modeling estimated direct and indirect associations of social integration and daily discrimination with glycosylated hemoglobin (HbA1c), high-density lipoprotein (HDL), C-reactive protein, and interleukin-6.

**Results:**

Social integration and daily discrimination were both significantly associated with self-esteem, which was in turn associated with HbA1c, HDL, and interleukin-6 levels. Social integration was indirectly associated with HbA1c, HDL, and interleukin-6 via self-esteem. Daily discrimination was directly associated with HbA1c, C-reactive protein, and interleukin-6 and was indirectly associated with HDL and interleukin-6 via self-esteem.

**Discussion and Implications:**

Findings identify social correlates of inflammation and cardiovascular risk and suggest that self-esteem may serve as a pathway for effects. Overall, results were somewhat mixed: Daily discrimination was directly associated with both self-esteem and 3 of the 4 biological markers of health; however, although social integration was strongly associated with self-esteem, it was only weakly and indirectly associated with biological health markers. Moreover, the indirect effects of daily discrimination on the biomarker outcomes—while significant—were notably smaller than its direct effects. Implications for theory, practice, and future research are discussed, including the need for further study of self-esteem and physical health across mid- and later life.

**Translational Significance:** Social integration and daily discrimination have known implications for health throughout the life course, yet the mechanisms for such effects are not fully understood. This study provides evidence that self-esteem acts as a pathway for social influences on health and suggests that interventions designed to bolster self-esteem may improve health outcomes and mitigate the harmful effects of social stressors in mid- and later life.

Social ties are influential for health and well-being throughout the life course (Umberson & Montez, 2010). This includes not only intimate and family relationships, but those of more distal ties such as social integration as well (Berkman et al., 2000). Indeed, social integration is associated with better physical, mental, and psychological well-being (Berkman et al., 2000; Stokes, 2019; Yang et al., 2016), whereas lacking such integration (i.e., social isolation) is associated with worse health and higher mortality risk (Holt-Lunstad et al., 2015; Yang et al., 2013, 2016). This study uses Keyes’ (1998) definition of social integration, a measure of perceived connectedness and belonging to a community. Feelings of social belonging may promote greater self-esteem and, in so doing, benefit health (Stokes, 2019).

Not all relationships are beneficial for health: Positive and supportive relationships promote better health, whereas negative and straining relationships undermine health (Birditt et al., 2018; Lund et al., 2014; Pascoe & Smart Richman, 2009; Umberson & Montez, 2010). Indeed, negative social interactions can be harmful, even when these interactions are with peripheral social ties. For instance, daily discrimination—a measure of perceived experiences of poor or unfair treatment from others—has been repeatedly linked with worse psychological and physical well-being in observational, longitudinal, and experimental studies (Pascoe & Smart Richman, 2009; Schmitt et al., 2014). Daily discrimination has even been associated with elevated mortality risk (Farmer et al., 2019). It is not merely strain in one’s close relationships that can damage health and well-being; everyday discrimination in the workplace, neighborhood, or elsewhere carries real harms, as well (Stokes & Moorman, 2020). Moreover, some evidence indicates that social integration and discrimination may be particularly impactful for older adults, and especially the oldest-old (Birditt et al., 2018; Charles, 2010; Stokes, 2019), making mid- and later life a key period for inquiry.

Although links between both social integration and perceived discrimination with adults’ health have been well-established, the mechanisms for these effects remain less clear (Farmer et al., 2019; Uchino et al., 2012; Yang et al., 2013, 2016). This study builds upon recent theoretical and empirical work concerning social determinants of health in mid- and later life (Cohen, 1988, 2004; Kim & Thomas, 2019; Thoits, 2011) and, in particular, examines self-esteem as a potential mediator linking social integration and daily discrimination with biological markers of inflammation and cardiovascular health (glycosylated hemoglobin [HbA1c], high-density lipoprotein [HDL], C-reactive protein [CRP], and interleukin-6 [IL-6]). Findings will contribute to the growing literature on how social experiences outside close personal relationships may get “under the skin” to affect health throughout the life course.

## The Role of Self-Esteem

Previous research has revealed the importance of negative aspects of mental health (e.g., depression) for physical health and mortality, including in analyses of mechanisms linking social ties and stressors to health (Berkman et al., 2000; Farmer et al., 2019; Holt-Lunstad et al., 2015; Pascoe & Smart Richman, 2009; Schulz et al., 2000). Increasingly, however, the implications of positive emotions for health outcomes have become a focus of social and psychological research (Boehm & Kubzansky, 2012; Kim & Thomas, 2019; Steptoe et al., 2015). Indeed, recent studies have revealed links between self-esteem and self-rated health, functional health, and reported health problems (Orth et al., 2012; Reitzes & Mutran, 2006; Stinson et al., 2008). Self-esteem may even be linked with neuroendocrine responses to stressors (Seeman et al., 1995). Moreover, self-esteem is not a stable trait throughout adulthood, but rather can be bolstered or impaired by social relations and interactions throughout the life course (Stokes, 2019; Thoits, 2011).

Importantly, the effects of positive well-being on health outcomes appear to be independent of the influence(s) of poor mental health (Ostir et al., 2001; Ryff et al., 2006; Steptoe et al., 2005). Overall, evidence suggests a protective role of self-esteem for adults’ health, which may even be of heightened importance with age (Birditt et al., 2018; Boehm & Kubzansky, 2012; Steptoe et al., 2015). This also implies that certain positive aspects of well-being, such as self-esteem, may be important yet understudied as mechanisms linking social ties with biological health outcomes across the life course (Kim & Thomas, 2019; Uchino et al., 2012).

This study takes a psychosocial process approach to analyzing social ties and health (Cohen, 1988). In particular, Cohen’s (1988, 2004) identity and self-esteem model asserts that social integration influences health by promoting positive psychological states (e.g., sense of identity and self-esteem); these in turn affect both health behaviors and physiological response mechanisms. Lacking social integration (i.e., isolation), on the other hand, may harm positive psychological states and reduce adults’ sense of control and self, with detrimental implications for behavioral and physiological factors (Cohen, 1988, 2004).

Additionally, this study uses Thoits’ (2011) theoretical framework of mechanisms linking social ties to health, which notes that self-esteem in particular is derived from individuals’ own judgments of their role performances, that is, from individuals’ belief that others value and appreciate them—or not. As a result, self-esteem may be eroded by perceived slights, social rejection, or discriminatory interactions (Charles, 2010; Schmitt et al., 2014; Thoits, 2011). Self-esteem, then, is likely to play an intermediary role: affected by both social integration and everyday discrimination, and thus linking them with biological health outcomes in mid- and later life, for better *and* for worse.

Recent empirical evidence is limited but offers general support for this theoretical approach. For instance, self-esteem can be enhanced by perceptions of social integration (Stokes, 2019), yet is undercut by perceived daily discrimination (Schmitt et al., 2014; Stokes, 2019). In turn, self-esteem may be influential for trajectories of physical and biological health (Orth et al., 2012; Reitzes & Mutran, 2006; Seeman et al., 1995; Stinson et al., 2008). However, few studies have explicitly positioned self-esteem as a pathway linking social ties and health, particularly among samples of aging adults. Indeed, only one recent study explicitly modeled mediation, with mixed results: Kim and Thomas (2019) found that self-esteem mediated the association between social support and mental health, but not between social support and CRP levels. The present study builds upon this prior literature and examines whether self-esteem may mediate the associations of both social integration and daily discrimination with biological markers of inflammation and cardiovascular health among a sample of midlife and older adults.

## Biomarkers

This study analyzes four biological markers of health: HbA1c, HDL, CRP, and IL-6 (Weinstein et al., 2017). Self-reported health is a robust predictor of all-cause mortality (Idler & Benyamini, 1997), yet self-reported measures of health may be subject to error or bias based in part on participants’ emotional states (Whitehead & Bergeman, 2016). Additionally, biological markers also allow for an assessment of specific physiological pathways whereby social ties and/or self-esteem may get “under the skin” to undermine—or promote—adults’ health.

HbA1c and HDL are measures of metabolic and cardiovascular health, as well as predictors of cardiovascular disease and mortality (Ahn & Kim, 2016; Khaw et al., 2004). Both are also responsive to health behaviors (Honda et al., 2014), which may be related not only with social integration and daily discrimination but with self-esteem as well (Boehm & Kubzansky, 2012; Cohen, 2004; Steptoe et al., 2009). CRP and IL-6 have likewise been associated with a host of age-related health conditions, including cardiovascular disease, arthritis, type II diabetes, frailty, and functional decline (Kiecolt-Glaser et al., 2010). These two biomarkers are both measures of inflammation and immune system function and are particularly sensitive to experiences of chronic stress (Kiecolt-Glaser et al., 2010). Daily discrimination is a known psychosocial stressor, yet social (dis)connectedness and poor self-esteem may also be experienced as stressors or exacerbate stress responses (O’Donnell et al., 2008; Stokes, 2019; Yang et al., 2013). Furthermore, insofar as discrimination and/or social (dis)connectedness undermine self-esteem, individuals may lose a psychosocial coping mechanism that protects against harmful effects of *other* chronic stressors (Stokes, 2019; Thoits, 2011). Taken together, these biological markers offer information on distinct physiological pathways whereby social experiences and self-esteem may influence adults’ health.

## Study Aims

The present study examines associations of social integration and daily discrimination with four biological markers of health among a probability-based sample of midlife and older adults. Moreover, this study takes a psychosocial process approach to health, specifically anticipating that (H1) social integration and daily discrimination will be significantly associated with self-esteem; (H2) self-esteem will in turn be associated with biological markers of health; and (H3) the associations of social integration and daily discrimination with biological markers of health will be at least partially mediated by self-esteem. Due to the age range of participants, I also test whether (H4) any associations of interest are stronger among older respondents. Results will contribute to the literature concerning social determinants of health across the life course and will highlight potentially important mechanisms for the health effects of social ties beyond one’s close relationships in mid- and later life.

## Method

Data for this study came from the National Survey of Midlife Development in the United States (MIDUS) Refresher and the MIDUS Refresher Biomarker Project. Collected between 2011 and 2014, the MIDUS Refresher is a national probability sample of adults aged 25–74 and was designed to parallel the baseline sample from the original 1995–1996 MIDUS I study (Ryff et al., 2016). Participants were selected using a combination of a random-digit dial (RDD) landline sampling frame, a list frame targeted to decadal age brackets, and an RDD cell phone sampling frame (Ryff et al., 2016). Information was gathered from respondents using both a telephone interview and a self-administered questionnaire (SAQ). Of those contacted for the telephone interview, 59% (*N* = 3,577) agreed to participate. Among the participants who completed the phone interview, 73% (*N* = 2,598) also completed the SAQ (Ryff et al., 2016). Participants who responded to both the phone interview and the SAQ were then eligible for the MIDUS Refresher Biomarker Project, and 29% (*N* = 746) of eligible participants completed the biomarker data collection (Weinstein et al., 2017). Biomarker Project participants were significantly older and less likely to be widowed than were MIDUS participants who did not complete the biomarker data collection, and also reported higher average income, greater educational attainment, lower levels of neuroticism, higher alcohol consumption, and lower rates of current smoking than main sample participants. Descriptive statistics for the MIDUS main sample and Biomarker Project subsample are reported in Supplementary Table 1.

Biomarker data collection proceeded from 2012 to 2016, and all assessments were made during a 24-hr overnight stay at one of the three regional Clinical Research Units (CRUs), depending on participants’ location (Weinstein et al., 2017). The biomarker measures used in this study were taken from a fasting blood draw, administered in the morning after participants’ overnight stay at the CRU (Weinstein et al., 2017). The final analytic sample includes the 746 MIDUS Refresher participants who completed the phone interview, SAQ, and biomarker data collection (Weinstein et al., 2017).

### Measures

#### Biomarkers

##### *Glycosylated hemoglobin*.—

HbA1c was included as a measure of glucose metabolism. HbA1c was measured at Meriter Labs in Madison, WI, using a Roche Cobas Analyzer to assay fresh whole blood drawn from a fasting blood sample (Weinstein et al., 2017). In order to correct for significant positive skew, HbA1c was transformed using the negative inverse (−1/X).

##### *High-density lipoprotein*.—

HDL levels were included as a measure of cardiovascular health. HDL was measured at Meriter Labs in Madison, WI, using a Roche Cobas Analyzer to assay frozen serum taken from a fasting blood draw (Weinstein et al., 2017). A logarithmic transformation was used to correct for significant positive skew.

##### *C-reactive protein*.—

CRP, an acute-phase protein, was included as a measure of systemic inflammation. CRP assays were performed at the Tracy Lab at the University of Vermont. CRP was initially measured using a particle-enhanced immunonephelometric assay on plasma; samples falling below the assay range were then reexamined by immunoelectrochemiluminescence using a high-sensitivity assay (Weinstein et al., 2017). From 2016 onward, the latter assay was used for all samples, using serum rather than plasma; corrections were applied to ensure consistency across assay methods (Weinstein et al., 2017). In order to correct for significant positive skew, a logarithmic transformation was performed.

##### *Interleukin-6*.—

IL-6, a proinflammatory cytokine, was included as a measure of systemic inflammation. IL-6 was assayed using frozen serum taken from a fasting blood draw sample at the MIDUS Biocore Laboratory at the University of Wisconsin, Madison. The assay was performed using a Quantikine High-Sensitivity ELISA Kit (Weinstein et al., 2017). In order to correct for significant positive skew, a logarithmic transformation was applied.

#### Predictors of interest

##### Self-esteem.—

Self-esteem was measured using a seven-item mean-score scale (α = 0.78; Rosenberg, 1965). Participants were asked to respond on a scale from 1 (*Strongly agree*) to 7 (*Strongly disagree*) to statements such as “I take a positive attitude toward myself” and “On the whole, I am satisfied with myself.” Higher values indicated better self-esteem.

##### Social integration.—

Social integration was measured using a three-item mean-score scale (α = 0.77; Keyes, 1998). Participants were asked to respond on a scale from 1 (*Strongly agree*) to 7 (*Strongly disagree*) to questions such as “I feel close to other people in my community” and “My community is a source of comfort.” Higher values indicated greater social integration.

##### Daily discrimination.—

Participants were asked a series of nine questions concerning how often they experienced different forms of all-cause day-to-day discrimination (Williams et al., 1997). Sample items include “[How often] are you treated with less respect than other people?” and “[How often do] people act as if they think you are not smart?” Response options ranged from 1 (*Never*) to 4 (*Often*) (α = 0.92). There was substantial skew in the discrimination scale, largely due to the prevalence of “never” as a response. Therefore, perceived daily discrimination was transformed using the natural logarithm.

##### Age.—

Age was measured in years (range = 25–76) and was mean-centered for analysis.

#### Covariates

A series of control variables were included to protect against potential confounding and to ensure the robustness of significant associations concerning the measures of interest. These covariates accounted for participants’ demographic characteristics, as well as their health and health behaviors. Demographic measures included participants’ race, ethnicity, gender, income, marital status, parental status, employment status, educational attainment, and neuroticism (Farmer et al., 2020; Kessler et al., 1999; Montez et al., 2011; Stokes, 2019, 2020; Umberson et al., 2013). Health and health behaviors included history of diabetes, experience of depression, caffeine consumption, alcohol consumption, smoking behavior, use of cholesterol, antihypertensive, and antidepressive medications, and exercise (Ahn & Kim, 2016; Kiecolt-Glaser & Glaser, 2002; Stokes, 2020; Wang et al., 2008).

Participants’ race was measured using dichotomous indicators for *white* (reference) and *nonwhite*, whereas ethnicity was measured using dichotomous indicators for *not Hispanic* (reference) and *Hispanic*. Gender was measured using dichotomous indicators for *male* (reference) and *female*. *Income* was measured as total pre-tax income in the last calendar year in U.S. dollars and was standardized for analysis. Marital status was measured using dichotomous indicators for *married* (reference), *divorced/separated, widowed*, and *never married*. Parental status was measured using dichotomous indicators for *has children* (reference) and *does not have children*. Employment status was measured using dichotomous indicators for *employed* (reference), *unemployed*, *retired*, and *other employment status* (e.g., full-time student, homemaker, and permanently disabled). Educational attainment was measured using dichotomous indicators for *high school or less*, *some college* (reference), *college degree*, and *education beyond college*. *Neuroticism* was measured using a four-item mean-score scale ranging from 1 (*Lowest*) to 4 (*Highest*) (Lachman & Weaver, 1997).

History of diabetes was measured using dichotomous indicators for *never had diabetes* (reference), *history of borderline diabetes,* and *history of diabetes*. *Experience of depression* was measured using a dichotomous indicator of whether a participant reported feeling sad or depressed for 2 or more weeks during the past 12 months. *Caffeine consumption* was measured as a summary score scale of the number of servings of caffeinated coffee, caffeinated tea, and other caffeinated beverages participants reported consuming on an average day. *Alcohol consumption* was measured as the number of days in the past month participants reported drinking at least one alcoholic beverage and ranged from 1 (*Never*) to 6 (*Every day*). *Smoking* was measured using dichotomous indicators for *never smoked* (reference), *former smoker*, and *current smoker*. *Use of cholesterol medication*, *use of antihypertensive medication*, and *use of antidepressive medication* were all measured as dichotomous (Yes/No) self-reports from participants. *Exercise* was measured as a dichotomous self-report concerning whether participants engaged in any regular exercise or activity for 20 min or more at least 3 times per week.

There is some debate as to whether health and health behavior covariates should be included in analyses of health outcomes (Hebert et al., 2008), as these covariates may not account for potential confounding, but may instead act as mediators and thereby artificially reduce significant findings. However, not all health and health behavior measures are causal consequences of focal predictors such as self-esteem, yet they are important to account for when examining biomarker outcomes. For instance, history of diabetes is not only a highly significant predictor of HbA1c, but also reflects a combination of genetic and accumulated behavioral and other risk factors that temporally precede participants’ current psychological well-being (Busch & Hegele, 2001; Choi & Shi, 2001; Wild & Byrne, 2006). Given that HbA1c will by definition be higher among diabetics, it may thus be more appropriate to examine links between self-esteem and HbA1c after accounting for such differences, rather than pooling all individuals together; in other words, some health and behavioral covariates may be potential mediators, while others may be confounders or even suppressors (Hebert et al., 2008). Moreover, while psychological states such as self-esteem may influence health outcomes in part via behavioral pathways, there is also accumulating evidence that such health effects may be the result of physiological or biological response mechanisms (Boehm & Kubzansky, 2012; Cohen, 2004; Steptoe et al., 2009). Likewise, stressors such as everyday discrimination may harm health through both behavioral and physiological response processes (Cohen, 1988; Farmer et al., 2019; Pascoe & Smart Richman, 2009). Accounting for health and health behaviors as covariates allows for an assessment of the health impacts of the focal predictors, beyond what may be explained by behavioral pathways.

In order to examine these various possibilities empirically, sensitivity analyses were examined that (a) excluded all health and health behavior measures from the final model, and (b) excluded all health and health behavior measures *except history of diabetes* from the final model. Both sensitivity analyses produced results that were very similar to those presented below, with a few key differences. First, in both models, self-esteem became a significant predictor of CRP and a significant mediator of the indirect association between social integration and CRP. Other significant results of interest were largely unchanged. This indicates that the health and health behavior measures may partially mediate the associations of interest in this study, particularly for CRP, yet did not have a sizeable influence on the overall results. The one major exception concerns the results for self-esteem and HbA1c in the model that excluded all health and behavior measures, including history of diabetes. In this model, discrimination was a much stronger predictor of HbA1c, while self-esteem was reduced to nonsignificance (and therefore did not mediate any significant indirect effects). In this case, history of diabetes appears to be a suppressor: There was no clear association between self-esteem and HbA1c when all sample participants were averaged together, but there was a significant association once history of diabetes was accounted for. Full results from these sensitivity analyses are available from the author on request.

### Analytic Strategy and Missing Data

The majority of cases (76.7%) had complete data for all measures included in the final analysis. The item with the greatest amount of missingness was income, for which 8.6% of valid cases were missing data. Missing data diagnostics were performed, with no clear pattern of missingness detected. Therefore, full information maximum likelihood (FIML) was used to address missing data and protect against potential bias from listwise deletion (Allison, 2003).

Structural equation modeling (SEM) was used to address the present research questions. SEM allows for simultaneous estimation of multiple outcomes, including mediators; allows outcome variables to covary with one another; and allows for explicit estimation of direct and indirect effects, along with their statistical significance. All covariates were included in the analysis and were used as control measures in the equations for all endogenous variables (i.e., for both self-esteem and the four biomarker outcomes). Data management and analysis were performed using Stata/SE version 16.

## Results

### Descriptive Results

Descriptive statistics for the variables of interest are reported in Table 1. In general, participants reported feeling relatively integrated into their communities (mean = 4.79 on the seven-point scale), and perceived daily discrimination was quite moderate overall (mean = 1.45 on the four-point scale), with the largest proportion of participants reporting no discrimination (38.89%). Likewise, participants reported fairly high self-esteem, averaging 5.45 on the seven-point scale. Participants’ biomarkers were within normal ranges, as well: HbA1c levels averaged 5.64%, slightly below the 5.7% cutoff for concern of borderline or prediabetes (Mayo Medical Laboratories, 2018). HDL levels averaged 59.7 mg/dL, well above the 40 mg/dL cutoff for concern among men, and the 50 mg/dL cutoff for concern among women (Mayo Medical Laboratories, 2018). CRP averaged 2.78 μg/mL, well below the cutoff for concern of 8 μg/mL (Mayo Medical Laboratories, 2018). Lastly, IL-6 levels averaged 2.71 pg/mL, comparable to previous studies of proinflammatory cytokines in midlife and well below the cutoff for concern of 5 pg/mL (Mayo Medical Laboratories, 2018; Nersesian et al., 2018).

**Table 1.** Descriptive Statistics, MIDUS Refresher and Biomarker Project (*N* = 746)

Variables of interest	Mean (*SD*), or %
HbA1c^a^	5.64 (1.00)
HDL^a^	59.17 (19.83)
CRP^a^	2.78 (5.27)
IL-6^a^	2.71 (2.35)
Self-esteem	5.45 (1.08)
Social integration	4.79 (1.33)
Daily discrimination^a^	1.45 (0.54)
Age	51.62 (13.60)

*Note:* CRP = C-reactive protein; HbA1c = glycosylated hemoglobin; HDL = high-density lipoprotein; IL-6 = interleukin-6.

^a^Raw statistics presented; variable transformed for analysis.

### Analytic Results

The truncated results of the final structural equation model (SEM) are presented in Table 2. Full results of the SEM are available in Supplementary Table 2. All covariates were included in all five equations that were simultaneously estimated, in order to protect against potential confounding.

**Table 2.** Structural Equation Model Concerning Social Integration and Discrimination, Self-Esteem, and Biological Markers of Health Among Midlife and Older Adults in the United States (*N* = 746)

	Self-esteem	HbA1c^a^	HDL^a^	CRP^a^	IL-6^a^
*Direct effects*	*B* (*SE*)	*B* (*SE*)	*B* (*SE*)	*B* (*SE*)	*B* (*SE*)
Self-esteem	—	−0.08* (0.04)	0.04** (0.01)	−0.08 (0.05)	−0.09** (0.03)
Social integration	0.17*** (0.02)	0.02 (0.02)	−0.00 (0.01)	−0.02 (0.03)	−0.00 (0.02)
Daily discrimination	−0.31** (0.10)	0.31** (0.10)	−0.04 (0.03)	0.48*** (0.13)	0.19* (0.08)
Age	−0.01^†^ (0.00)	0.01* (0.00)	0.003* (0.00)	0.01 (0.00)	0.02*** (0.00)
*Indirect effects*					
Social integration → Self-esteem		−0.01* (.01)	0.01** (0.00)	−0.01 (0.01)	−0.02** (0.01)
Daily discrimination → Self-esteem		0.02^†^ (.01)	−0.01* (0.01)	0.02 (0.02)	0.03* (0.01)
*Model fit*	Self-esteem	HbA1c^a^	HDL^a^	CRP^a^	IL-6^a^
*R*^2^	40.41%	37.60%	34.09%	17.84%	32.75%
Log-likelihood^a^	−17,340.96				
χ ^2a^	1,818.45***				

*Notes:* CRP = C-reactive protein; HbA1c = glycosylated hemoglobin; HDL = high-density lipoprotein; IL-6 = interleukin-6; SE = standard error. Models were adjusted for race/ethnicity, gender, income, marital status, parental status, employment status, educational attainment, neuroticism, history of diabetes, experience of depression, caffeine consumption, alcohol consumption, smoking behaviors, use of cholesterol medication, use of antihypertensive medication, use of antidepressive medication, and exercise.

^a^Log-likelihood and χ ^2^ are model-based measures of goodness-of-fit and therefore apply to the model as a whole rather than to any specific outcome measure.

^†^ *p <* .10, *p < .05, **p < .01, ***p < .001.

Self-esteem was significantly predicted by both social integration (*B* = 0.17, *p* < .001) and perceived daily discrimination (*B* = −0.31, *p* < .01), as anticipated by Hypothesis 1. However, there was no significant age trajectory for self-esteem (*B* = −0.01, *p* > .05). Among covariates, female gender (*B* = 0.27, *p* < .001), being unemployed (*B* = −0.61, *p* < .001), neuroticism (*B* = −0.65, *p* < .001), experiencing depression (*B* = −0.39, *p* < .001), being a former (*B* = 0.25, *p* < .01) or current smoker (*B* = 0.38, *p* < .01), and taking antidepressive medications (*B* = −0.22, *p* < .05) were all significantly associated with self-esteem.

Concerning the biomarker outcomes, self-esteem was significantly associated with lower HbA1c (*B* = −0.08, *p* < .05), higher HDL (*B* = 0.04, *p* < .01), and lower IL-6 (*B* = −0.09, *p* < .01), as anticipated by Hypothesis 2. Self-esteem was not significantly associated with CRP (*B* = −0.07, *p* > .05). Furthermore, HbA1c (*B* = 0.01, *p* < .05), HDL (*B* = 0.003, *p* < .01), and IL-6 (*B* = 0.02, *p* < .001) all increased significantly with age. Social integration was not significantly directly associated with any of the biomarker outcomes, while perceived daily discrimination was significantly directly associated with higher HbA1c (*B* = 0.31, *p* < .01), CRP (*B* = 0.48, *p* < .001), and IL-6 (*B* = 0.19, *p* < .05) levels.

Furthermore, mediation was explicitly modeled and tested for significance. The final analysis revealed significant indirect associations of social integration with HbA1c (*B* = −0.01, *p <* .05), HDL (*B* = 0.01, *p* < .01), and IL-6 (*B* = −0.02, *p* < .01) levels via self-esteem, as well as significant indirect associations of daily discrimination with lower HDL (*B* = −0.01, *p* < .05) and higher IL-6 (*B* = 0.03, *p <* .05) via self-esteem. These significant indirect effects are in keeping with Hypothesis 3 that self-esteem would mediate the effects of social integration and/or daily discrimination on the biomarker outcomes. Supplementary Figure 1 displays the significant pathways detected in the final SEM analysis.

Among covariates, HbA1c was significantly predicted by history of borderline diabetes (*B =* 0.84, *p* < .001) and diabetes (*B* = 1.65, *p* < .001). HDL was significantly predicted by nonwhite race (*B* = 0.07, *p* < .05), female gender (*B* = 0.24, *p* < .001), having a college degree (*B* = 0.08, *p* < .01), alcohol consumption (*B* = 0.07, *p* < .05), being a current smoker (*B* = −0.08, *p* < .05), and exercise (*B* = 0.10, *p* < .001). CRP was significantly predicted by female gender (*B* = 0.29, *p* < .001), having a college degree (*B* = −0.24, *p <* .05) or education beyond college (*B* = −0.36, *p* < .01), neuroticism (*B* = −0.25, *p* < .01), history of diabetes (*B* = 0.34, *p* < .05), alcohol consumption (*B* = −0.06, *p* < .05), antihypertensive use (*B* = 0.30, *p* < .01), antidepressive use (*B* = 0.28, *p* < .05), and exercise (*B* = −0.21, *p* < .05). IL-6 was significantly predicted by nonwhite race (*B* = 0.17, *p* < .05), being retired (*B* = 0.27, *p* < .01), history of diabetes (*B* = 0.26, *p* < .01), alcohol consumption (*B* = −0.05, *p* < .01), antihypertensive use (*B =* 0.18, *p* < .01), and exercise (*B* = −0.20, *p* < .01). Interaction terms were examined between age and the other predictors of interest (not shown). No interactions were significant, failing to support Hypothesis 4.

## Discussion

The present study examined associations of social integration and daily discrimination with four biological markers of midlife and older adults’ health, and determined whether any such effects were mediated by self-esteem. The analysis revealed numerous associations between these social factors and biomarkers of health, both direct and indirect. These findings offer support for the identity and self-esteem model (Cohen, 1988) as well as Thoits’ (2011) framework linking social ties to health, and indicate that self-esteem may serve as an important pathway for the health effects of social integration and discriminatory interactions across mid- and later life. Additionally, findings underscore the harms of discrimination for health and suggest that the damages of negative social interactions may outweigh the benefits of positive ties (Baumeister et al., 2001; Lund et al., 2014; Pascoe & Smart Richman, 2009). However, findings were also mixed overall and indicate the need for caution in interpretation alongside the continued development of theoretical models concerning mechanisms linking social ties to health in mid- and later life (Uchino et al., 2012).

### Social Determinants of Self-Esteem and Health

Links between social relationships and health have been well-established (Berkman et al., 2000; Umberson & Montez, 2010), yet the precise mechanisms for such effects remain a topic of inquiry (Thoits, 2011; Uchino et al., 2012; Yang et al., 2013, 2016). This study took a psychosocial process approach to health and, using the identity and self-esteem model (Cohen, 1988, 2004) as well as Thoits’ (2011) framework linking social ties to health, posited that social integration and daily discrimination would have implications for biological health markers by way of their influences on self-esteem. Findings offered support for this framework overall: Social integration was associated with better self-esteem, while daily discrimination was associated with worse self-esteem. In turn, self-esteem was associated with three of the four biomarker outcomes and mediated multiple significant indirect effects. Thus, social integration and daily discrimination may influence biological health, in part, via their effects on self-esteem.

Associations between social integration and the biological health outcomes were notably weak, however. Not only were none of the direct associations significant, but the indirect associations—while statistically significant—provided scant evidence. In fact, post-hoc analyses (not shown) evaluating mediation using the inferential Baron and Kenny (1986) approach *failed* to meet all of the criteria. Thus, links between social integration and biological health in this study provide only meager support for hypothesized health effects and mediation.

The same was not true of daily discrimination, however. Although social integration and daily discrimination both stood out as strong predictors of self-esteem in this study (Schmitt et al., 2014), daily discrimination had much clearer associations with the measures of biological health. This suggests that stressors such as discrimination may undermine health more strongly than perceptions of belonging can support it (Baumeister et al., 2001; Lund et al., 2014). This appears particularly true in the present sample, given the relatively small coefficient sizes for significant indirect effects, in comparison with the much larger coefficients for the direct effects of daily discrimination on HbA1c, IL-6, and CRP levels.

Furthermore, chronic stressors such as everyday discrimination may be particularly harmful, as their repeated impacts accrue over time (Farmer et al., 2019). Indeed, contemporaneous reports of daily discrimination may reflect the accumulation of stressful experiences and discriminatory interactions throughout the life course (Dannefer, 2020; Kessler et al., 1999; Pascoe & Smart Richman, 2009). Such processes of differential exposure to stressors and cumulative dis/advantage may help to explain persistent racial disparities in cardiovascular health and mortality among American adults (Farmer et al., 2019, 2020; Williams et al., 1997; Williams & Mohammed, 2009). Future research using longitudinal life course data will be needed to determine whether contemporaneous reports of social integration and discrimination truly have unequal effects on inflammation and cardiovascular health, or whether perceptions of discrimination in mid- and later life reflect the long-term accumulation of chronic stressors and their harmful effects.

### The Intermediary Role of Self-Esteem

Research has increasingly explored the role of positive psychological and emotional well-being for adults’ health (Boehm & Kubzansky, 2012; Steptoe et al., 2015). However, an ongoing debate concerns the extent to which psychological factors truly mediate the effects of social connections on adults’ physical health outcomes (Uchino et al., 2012). The cross-sectional design of this study limits its ability to wade into this debate fully, yet the present findings are suggestive and may help inform future research that is designed to establish causality and mediation over time. First, as Uchino et al. (2012) note, the psychological mechanisms most commonly studied to date include anxiety, distress, and depression, although aspects of well-being such as positive affect have received a fair amount of research attention as well. Self-esteem, however, has remained a largely understudied component of well-being (Uchino et al., 2012), yet emerging evidence suggests that it may be an important psychological mechanism linking social ties to health (Kim & Thomas, 2019; Orth et al., 2012; Reitzes & Mutran, 2006; Stinson et al., 2008; Symister & Friend, 2003; Thoits, 2011). The present findings reinforce this view: Self-esteem emerged as a consistent predictor of the biological markers of health, in addition to being predicted by both social integration and daily discrimination. Furthermore, while self-esteem’s nonsignificant association with CRP coheres with prior research (Kim & Thomas, 2019), its significant association with IL-6 in this study indicates that inflammation may indeed be one pathway by which self-esteem affects health, particularly because CRP production itself is stimulated by IL-6 (Gabay & Kushner, 1999). Longitudinal studies examining self-esteem and markers of inflammation and cardiovascular health beyond CRP will be needed to determine whether pathways such as IL-6 truly link self-esteem with health over time. However, as theory continues to develop concerning mechanisms linking social experiences to physical health outcomes, this study suggests greater attention should be paid to self-esteem and the importance of “mattering” (Thoits, 2011; Uchino et al., 2012).

An additional debate occurring in the field concerns whether aspects of well-being such as self-esteem get “under the skin” through the promotion of better health behaviors versus through physiological or biological response mechanisms (Boehm & Kubzansky, 2012; Cohen, 2004; Steptoe et al., 2009). The present findings suggest that self-esteem may have direct links with biological health, specifically HbA1c, HDL, and IL-6. Although cross-sectional and correlational, these associations were robust to the inclusion of various health behaviors as covariates, including the experience of depression, alcohol consumption, smoking behaviors, and exercise, in addition to measures concerning the use of cholesterol medications, antihypertensive medications, and antidepressive medications. A supplemental analysis excluding these health behavior measures (not shown) revealed similar findings to those presented, with one major exception being a significant direct association between self-esteem and CRP. This suggests that health behaviors may partly—but not entirely—mediate or confound links between emotional and biological well-being. Furthermore, this highlights both cardiovascular health and systemic inflammation as biological pathways for the health effects of self-esteem. This coheres with prior research (Steptoe et al., 2009) and highlights the importance of promoting better psychological, emotional, *and* social well-being for any efforts aimed at improving health and longevity among the aging population. Indeed, the present findings imply that interventions designed to improve self-esteem among midlife and older adults may be instrumental for successful health promotion, alongside efforts to reduce exposure to social stressors such as discrimination.

### Implications for Health Across the Life Course

Although the present study analyzed cross-sectional data, the results imply long-term and cumulative ramifications for adults’ health and well-being (Dannefer, 2020). For example, experiences of discrimination are stratified by demographic characteristics, particularly race/ethnicity, with some individuals reporting much greater lifetime exposure to everyday discrimination than others (Farmer et al., 2019; Kessler et al., 1999; Pascoe & Smart Richman, 2009). These experiences compound over time, contributing to growing disparities in well-being, health, and mortality risk across the life course (Dannefer, 2020; Pascoe & Smart Richman, 2009; Williams & Jackson, 2005). Likewise, access to supportive and integrative communities varies across individuals and over time, with stable (or expanding) differences in social integration also contributing to life-span inequalities in well-being and health (Keyes, 1998; Stokes, 2019; Williams & Jackson, 2005). Both advantages and disadvantages may accumulate throughout the life course, with the end result of exacerbating health disparities with age.

Additionally, there is some evidence of feedback loops between social ties and well-being, as well as between well-being and physical health (Orth et al., 2012; Reitzes & Mutran, 2006; Steptoe et al., 2015; Stinson et al., 2008). That is, poor or straining ties can harm self-esteem, which in turn may impair adults’ ability to improve, renew, or initiate supportive social relationships (Kim & Thomas, 2019; Orth et al., 2012; Stinson et al., 2008). Likewise, low self-esteem can undermine physical health, yet poor physical health also contributes to declines in self-esteem throughout mid- and later life (Reitzes & Mutran, 2006; Steptoe et al., 2015). This study analyzed only one direction of this potential feedback loop, but the present findings—situated within the extant literature—are suggestive of a downward spiral whereby deficits in one arena may lead to growing damage to all three arenas over time. This further underscores the importance of interventions designed to bolster self-esteem among the aging population, which may help to halt this harmful cycle in its tracks.

## Limitations

This study retains a number of limitations. First, the data analyzed were cross-sectional, restricting the causal interpretation of findings. Future research analyzing longitudinal data with social, emotional, and biological marker measures will be needed to better assess causality and to examine the validity of the hypothesized feedback loop(s) noted previously. Moreover, the sample analyzed was highly selective, comprising only 29% of eligible MIDUS Refresher participants. For instance, 80% of sample participants were white, only 5% reported Hispanic ethnicity, and the majority (57%) had either a college degree or some education beyond college. Future research is needed to determine the generalizability of findings from this study to more diverse and representative samples. Lastly, the present sample was restricted to the age range of 25–74 at recruitment, limiting any examination of potential differences in effects among the oldest-old. Although there were no age-based differences in effects detected in this study, some evidence indicates that social integration and social stressors such as discrimination may be particularly impactful for the oldest-old (Birditt et al., 2018; Charles, 2010; Stokes, 2019). Future research with samples that encompass an even broader age range should further assess the validity of results presented here and explore potential age differences in effects, particularly among the oldest-old.

### Conclusions

Despite these limitations, the present study contributes to both theory and the empirical literature concerning the health effects of social ties and interactions in adulthood. Results indicated that social integration and daily discrimination were both significantly associated with biological markers of inflammation and cardiovascular health among midlife and older adults, and that these associations were at least partly mediated by self-esteem. Daily discrimination was also significantly directly linked with three of the four biological health markers. These results offer support for the identity and self-esteem model (Cohen, 1988, 2004), as well as Thoits’ (2011) framework linking social ties to health, and indicate that self-esteem may be an important, yet understudied, mechanism whereby social ties get “under the skin” to affect health throughout the life course (Boehm & Kubzansky, 2012; Kim & Thomas, 2019; Thoits, 2011). However, findings overall were somewhat mixed: Social integration was associated with biological markers of health very weakly and only indirectly. Moreover, the indirect associations of daily discrimination with biological markers of health—while significant—were notably smaller than its significant direct associations. Yet overall, this study establishes self-esteem as a potentially important pathway for the effects of social connectedness and interactions on health in mid- and later life and underscores the importance of reducing exposure to social stressors across the life course for the effective promotion of both positive well-being and better health among the aging population (Baumeister et al., 2001; Farmer et al., 2019; Lund et al., 2014; Pascoe & Smart Richman, 2009). Overall, the results of this study should inform future research and theory concerning potential mechanisms for social determinants of midlife and older adults’ health.

## Supplementary Material

igaa026_suppl_Supplementary_Material
