# Supplementary material for: Social Integration, Daily Discrimination, and Biological Markers of Health in Mid- and Later Life: Does Self-Esteem Play an Intermediary Role?
Source: Innov Aging. 2020 Jul 6;4(4):igaa026. doi: 10.1093/geroni/igaa026 (PMC7413616; doi:10.1093/geroni/igaa026)
Supplement: igaa026_suppl_Supplementary_Material [file igaa026_suppl_supplementary_material.docx]

Online Supplementary Material

Supplemental Figure 1. *Significant Pathways in SEM Analysis Concerning Social Ties, Self-Esteem, and Biological Markers of Health.*


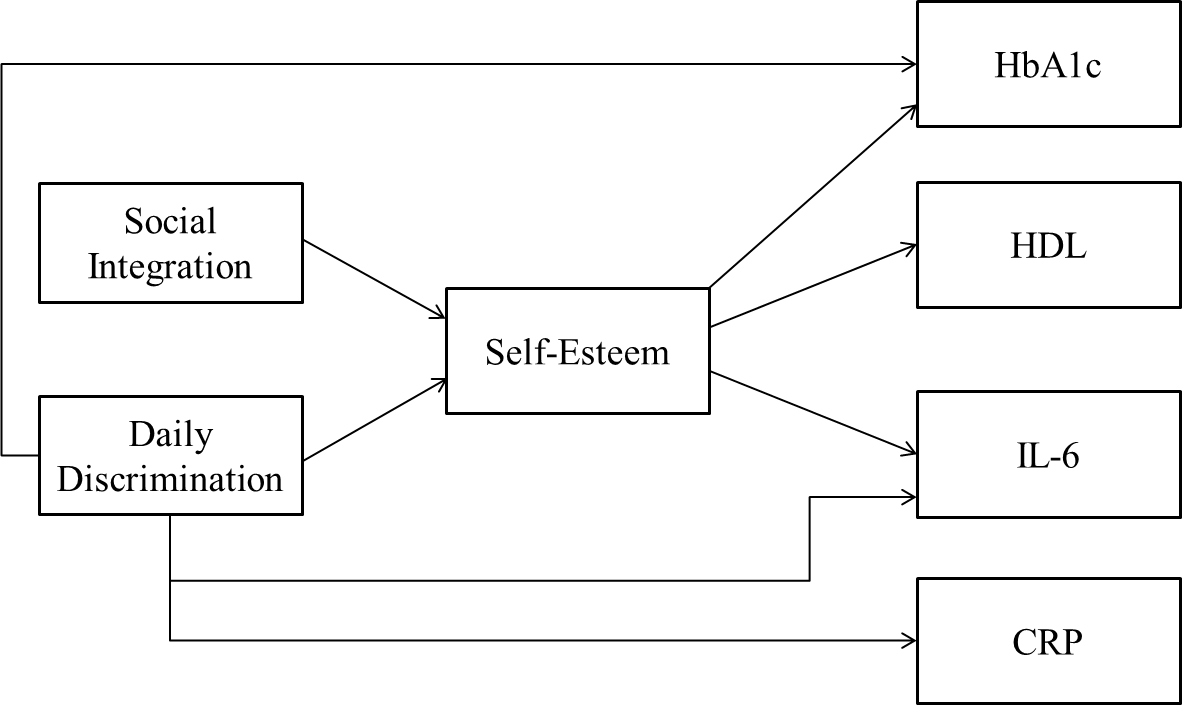


Supplemental Table 1. *Descriptive Statistics & Sample Selectivity, MIDUS Refresher Biomarker Project Subsample (N = 746) and MIDUS Refresher Non-Biomarker Participants (N = 2,831)*

|  | Biomarker Subsample | MIDUS Participants |  |
| --- | --- | --- | --- |
| *Variables of Interest* | Mean (SD), or % | Mean (SD), or % | P-value |
| HbA1c^a, b^ | 5.64 (1.00) | - | - |
| HDL^a,b^ | 59.17 (19.83) | - | - |
| CRP^a,b^ | 2.78 (5.27) | - | - |
| IL-6^a,b^ | 2.71 (2.35) | - | - |
| Self-esteem | 5.45 (1.08) | 5.45 (1.09) | - |
| Social integration | 4.79 (1.33) | 4.71 (1.31) | - |
| Daily discrimination^a^ | 1.45 (0.54) | 1.48 (.54) | - |
| Age | 51.62 (13.60) | 50.21 (14.56) | * |
| *Covariates* |  |  |  |
| Race: |  |  |  |
| *White* | 80.97% | 82.71% | - |
| *Nonwhite* | 19.03% | 17.29% | - |
| Ethnicity: |  |  |  |
| *Hispanic* | 4.56% | 5.90% | - |
| *Not Hispanic* | 95.44% | 94.10% | - |
| Gender: |  |  |  |
| *Male* | 50.13% | 47.58% | - |
| *Female* | 49.87% | 52.42% | - |
| Income^a^ | $56,638.16 ($48,840.76) | $49,774.13 ($49,004.80) | ** |
| Marital status: |  |  |  |
| *Married* | 64.87% | 63.77% | - |
| *Divorced/separated* | 15.75% | 15.37% | - |
| *Widowed* | 4.04% | 5.98% | * |
| *Never married* | 15.34% | 14.87% | - |
| Parental status: |  |  |  |
| *Has childrenas* | 77.88% | 79.97% | - |
| *Does not have children* | 22.12% | 20.03% | - |
| Employment status: |  |  |  |
| *Employed* | 67.63% | 66.20% | - |
| *Unemployed* | 4.48% | 3.39% | - |
| *Retired* | 21.24% | 20.45% | - |
| *Other* | 6.65% | 9.96% | ** |
| Educational attainment: |  |  |  |
| *High school or less* | 14.23% | 25.59% | *** |
| *Some college* | 28.59% | 31.26% | - |
| *College degree* | 26.71% | 22.30% | * |
| *Education beyond college* | 30.47% | 20.85% | *** |
| Neuroticism | 2.08 (0.66) | 2.16 (.63) | ** |
| History of diabetes^b^: |  |  |  |
| *No diabetes* | 87.13% | - | - |
| *Borderline diabetes* | 3.49% | - | - |
| *Diabetes* | 9.38% | - | - |
| Experienced depression^b^: |  |  |  |
| *Yes* | 23.46% | - | - |
| *No* | 76.54% | - | - |
| Caffeine consumption^b^ | 4.66 (4.13) | - | - |
| Alcohol consumption | 2.65 (1.51) | 2.27 (1.40) | *** |
| Smoking: |  |  |  |
| *Never smoked* | 61.93% | 56.27% | ** |
| *Former smoker* | 28.95% | 29.48% | - |
| *Current smoker* | 9.12% | 14.25% | *** |
| Cholesterol medication: |  |  |  |
| *Yes* | 26.32% | 26.85% | - |
| *No* | 73.68% | 73.15% | - |
| Anti-hypertensive medication^b^: |  |  |  |
| *Yes* | 34.05% | - | - |
| *No* | 65.95% | - | - |
| Anti-depressive medication^b^: |  |  |  |
| *Yes* | 16.35% | - |  |
| *No* | 83.65% | - |  |
| Exercise^b^: |  |  |  |
| *Yes* | 76.81% | - | - |
| *No* | 23.19% | - | - |

^a^Raw statistics presented; variable transformed for analysis. ^b^Data unavailable for the MIDUS main sample participants; measure drawn from the biomarker data set.

Supplemental Table 2. *Structural Equation Model Concerning Social Integration and Discrimination, Self-Esteem, and Biological Markers of Health Among Midlife and Older Adults in the United States (*N *= 746)*

|  | Self-Esteem | HbA1c^a^ | HDL^a^ | CRP^a^ | IL-6^a^ |
| --- | --- | --- | --- | --- | --- |
| *Direct effects of interest* | B (SE) | B (SE) | B (SE) | B (SE) | B (SE) |
| Self-esteem | - | -0.08* (.04) | 0.04** (.01) | -0.08 (.05) | -0.09** (.03) |
| Social integration | 0.17*** (.02) | 0.02 (.02) | -0.00 (.01) | -0.02 (.03) | -0.00 (.02) |
| Daily discrimination^a^ | -0.31** (.10) | 0.31** (.10) | -0.04 (.03) | 0.48*** (.13) | 0.19* (.08) |
| Age | -0.01^Ϯ^ (.00) | 0.01* (.00) | 0.003* (.00) | 0.01 (.00) | 0.02*** (.00) |
| *Indirect effects of interest* |  |  |  |  |  |
| Social integration 🡪 Self-esteem | | -0.01* (.01) | 0.01** (.00) | -0.01 (.01) | -0.02** (.01) |
| Daily discrimination^a^ 🡪 Self-esteem | | 0.02^Ϯ^ (.01) | -0.01* (.01) | 0.02 (.02) | 0.03* (.01) |
| *Covariates* |  |  |  |  |  |
| Nonwhite^b^ | 0.10 (.09) | 0.04 (.08) | 0.07* (.03) | -0.18 (.11) | 0.17* (.07) |
| Hispanic^c^ | 0.12 (.15) | -0.15 (.14) | -0.06 (.05) | 0.08 (.20) | -0.12 (.12) |
| Female^d^ | 0.27*** (.07) | -0.00 (.07) | 0.24*** (.02) | 0.29** (.09) | -0.05 (.06) |
| Income^a^ | 0.03 (.04) | -0.03 (.04) | -0.01 (.01) | 0.01 (.05) | -0.02 (.03) |
| Divorced/separated^e^ | 0.16^Ϯ^ (.09) | 0.17^Ϯ^ (.09) | -0.03 (.03) | 0.02 (.12) | 0.01 (.07) |
| Widowed^e^ | -0.04 (.17) | -0.17 (.16) | 0.09^Ϯ^ (.05) | -0.27 (.22) | -0.10 (.13) |
| Never married^e^ | -0.06 (.11) | -0.02 (.11) | -0.03 (.04) | 0.11 (.14) | 0.04 (.09) |
| Does not have children^f^ | -0.08 (.09) | -0.15^Ϯ^ (.08) | 0.03 (.03) | 0.09 (.12) | 0.05 (.07) |
| Unemployed^g^ | -0.61*** (.17) | -0.07 (.16) | 0.00 (.05) | 0.21 (.22) | 0.24^Ϯ^ (.14) |
| Retired^g^ | 0.17 (.10) | -0.09 (.10) | -0.03 (.03) | 0.05 (.13) | 0.27** (.08) |
| Other employment^g^ | 0.06 (.14) | -0.15 (.13) | 0.02 (.04) | -0.20 (.17) | -0.02 (.11) |
| High school or less^h^ | -0.12 (.10) | 0.15 (.10) | -0.06^Ϯ^ (.03) | 0.15 (.14) | 0.02 (.08) |
| College degree^h^ | -0.16^Ϯ^ (.09) | -0.14^Ϯ^ (.08) | 0.08** (.03) | -0.24* (.11) | -0.12^Ϯ^ (.07) |
| Education beyond college^h^ | -0.02 (.09) | -0.09 (.08) | 0.02 (.03) | -0.36** (.12) | -0.07 (.07) |
| Neuroticism | -0.65*** (.05) | -0.10^Ϯ^ (.05) | -0.00 (.02) | -0.25** (.07) | -0.06 (.05) |
| Borderline diabetes^i^ | 0.01 (.17) | 0.84*** (.16) | 0.00 (.06) | 0.18 (.22) | 0.06 (.14) |
| Diabetes^i^ | 0.05 (.12) | 1.67*** (.11) | -0.05 (.04) | 0.34* (.15) | 0.26** (.09) |
| Experienced depression | -0.39*** (.08) | -0.04 (.08) | -0.01 (.03) | 0.14 (.11) | 0.00 (.07) |
| Caffeine consumption | -0.01 (.01) | 0.00 (.01) | -0.00 (.00) | 0.00 (.01) | 0.01 (.01) |
| Alcohol consumption | 0.02 (.02) | -0.02 (.02) | 0.07*** (.01) | -0.06* (.03) | -0.05** (.02) |
| Former smoker^j^ | 0.25** (.07) | 0.12^Ϯ^ (.07) | 0.01 (.02) | 0.08 (.09) | 0.06 (.06) |
| Current smoker^j^ | 0.38** (.12) | 0.02 (.12) | -0.08* (.04) | 0.07 (.16) | 0.13 (.10) |
| Cholesterol medication use | 0.07 (.09) | 0.14^Ϯ^ (.08) | -0.03 (.03) | -0.17 (.11) | -0.00 (.07) |
| Anti-hypertensive use | 0.02 (.08) | -0.03 (.08) | -0.04^Ϯ^ (.03) | 0.30** (.10) | 0.18** (.06) |
| Anti-depressive use | -0.22* (.09) | -0.04 (.08) | 0.05^Ϯ^ (.03) | 0.28* (.12) | 0.09 (.07) |
| Exercise | 0.07 (.08) | -0.04 (.07) | 0.10*** (.02) | -0.21* (.10) | -0.20** (.06) |
| *Covariances* | Self-Esteem | HbA1c^a^ | HDL^a^ | CRP^a^ | IL-6^a^ |
| HDL^a^ | - | -0.03** (.01) | - | - | - |
| CRP^a^ | - | 0.08* (.03) | -0.06*** (.01) | - | - |
| IL-6^a^ | - | 0.02 (.02) | -0.04*** (.01) | 0.40*** (.03) | - |
| *Model fit* | Self-Esteem | HbA1c^a^ | HDL^a^ | CRP^a^ | IL-6^a^ |
| R^2^ | 40.41% | 37.60% | 34.09% | 17.84% | 32.75% |
| Log-likelihood^k^ | -17340.96 | | | | |
| χ^2 a^ | 1818.45*** | | | | |

^a^Tranformed variable. ^b^Reference group is White. ^c^Reference group is Not Hispanic. ^d^Reference group is Male. ^e^Reference group is Married. ^f^Reference group is Has children. ^g^Reference group is Employed. ^h^Reference group is Some college. ^i^Reference group is No history of diabetes. ^j^Reference group is Never smoked. ^k^Log-likelihood and χ^2^ are model-based measures of goodness-of-fit, and therefore apply to the model as a whole rather than to any specific outcome measure.

^Ϯ^ *p <* .10, * *p <* .05, ** *p <* .01, *** *p <* .001
